# Peptide-to-Protein Data Aggregation Using Fisher’s Method Improves Target Identification in Chemical Proteomics

**DOI:** 10.1021/acs.analchem.5c08021

**Published:** 2026-04-22

**Authors:** Hezheng Lyu, Hassan Gharibi, Zhaowei Meng, Bohdana Sokolova, Susanna Lundström, Xuepei Zhang, Roman A. Zubarev

**Affiliations:** † Division of Physiological Chemistry I, Department of Medical Biochemistry and Biophysics, 27106 Karolinska Institutet, Stockholm 171 65, Sweden; ‡ Biomotif AB, Täby 183 48, Sweden; § Swedish National Infrastructure for Biological Mass Spectrometry (BioMS), Stockholm 17177, Sweden; ∥ Chemical Proteomics Unit, Science for Life Laboratory (SciLifeLab), Stockholm 171 65, Sweden; ⊥ Single Cell Proteomics Facility, Department of Medical Biochemistry and Biophysics, Karolinska Institutet, Stockholm 171 65, Sweden; # Department of Pharmacological & Technological Chemistry, I.M. Sechenov First Moscow State Medical University, Moscow 119146, Russia

## Abstract

Protein-level statistical tests in proteomics, aimed at obtaining p-values, are conventionally made on protein abundances aggregated from peptide data. This integral approach overlooks peptide-level heterogeneity and ignores important information coded in individual peptide data, while protein p-values can also be obtained by Fisher’s method of combining peptide p-values using chi-square statistics. Here, we test this latter approach across diverse chemical proteomics data sets based on assessments of protein expression, solubility, and protease accessibility. Using the top four peptides ranked by their p-values consistently outperformed protein-level analysis and avoided biases introduced by the inclusion of deviant peptides or the imputation of missing peptide values. Fisher’s method provides a simple and robust strategy, improving identification of regulated/shifted proteins in diverse proteomics assays.

## Graphical abstract

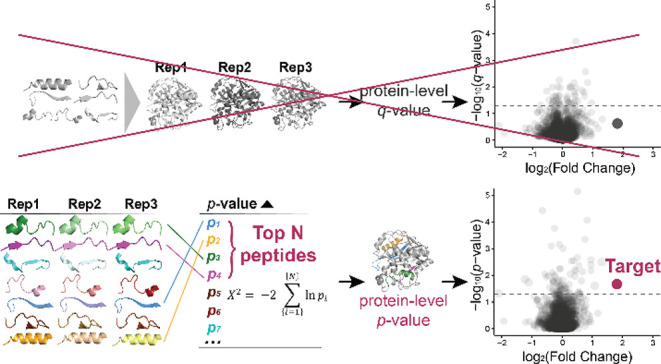

## Introduction

Differential expression analysis between two groups of samples is the most common type of analysis performed in proteomics. One of the most important steps is the determination of p-value, which is the a priori probability for protein abundance to differ by the observed or higher value. The Student’s *t* test applied to data from several replicate analyses is commonly used for such purpose. In proteomics, the p-values obtained for individual proteins need to be corrected for multiple hypotheses using, e.g., Bonferroni or Benjamini–Hochberg correction.^1^ Often, few (if any) statistically significant proteins remain after stringent correction, even though many protein abundances seem to change. This gives rise to the false negative problem in, e.g., drug discovery, where multiple drug targets need to be identified and characterized.

Part of the problem is in the suboptimal data processing. Search engines commonly used in bottom-up proteomics, such as Proteome Discoverer,^2^ MaxQuant,^3^ and Mascot,^4^ extract first peptide abundances and then aggregate them into protein abundance. This is done by the algorithms that are often not transparently documented. Sometimes, the abundances of peptides belonging to the same protein are simply summed together. The peptides attributed to a given protein by mistake or those with highly fluctuating abundances (e.g., due to post-translational modifications, PTMs) will also contribute to the result, reducing its statistical significance. If the peptide data are missing in some replicates, they are frequently imputed by arbitrary values. The aggregated protein abundance can therefore be a mixture of reliable and questionable peptide data, which leads to higher (poorer) p-values than justified.

There are approaches to filter peptide data before aggregating them into proteins, such as the Diffacto technique.^5^ These approaches are based on investigating peptide behavior in different samples through covariation analysis and rejecting the deviating peptides. Such techniques work particularly well when several nonreplicate samples (e.g., obtained by different treatments) are present in the data set, and require substantial bioinformatic efforts. A simpler and more straightforward approach could be to use Fisher’s formula for merging p-values of independent series of measurements.^6^ As unique peptides in proteomics are statistically independent entities measured in several replicates, a *t* test can be performed for each peptide. Thus, we hypothesized that it should be possible to integrate with advantage these peptide data into a protein p-value via Fisher’s formula.

To test this hypothesis, we reanalyzed using different peptide-to-protein strategies several chemical proteomics data sets obtained in our lab by a variety of proteomics techniques. This includes the data sets assessing the changes in protein expression (FITExP^7^ and ProTargetMiner^8^), and solubility (PISA^9^ and OPTI-PISA^10^). As the results clearly demonstrated the superior performance of Fisher’s-based technique over the conventional approach, the optimal analytical strategy was validated on the data sets assessing the protease accessibility of protein domains (AFDIP^11^ and HOLSER^12^).

## Methods

### Data Processing and Abundance Normalization

Each data set was composed of several raw LC-MS/MS data files of tryptic peptide mixtures with tandem mass tag (TMT) multiplexing. The data were processed with either MaxQuant (MQ) or Proteome Discoverer (PD) analysis programs, or both. In the latter case, both MQ- and PD-obtained results were used in further analysis. Peptide-level outputs were used for Fisher’s analysis, while protein-level outputs served for comparison with Fisher’s results. Peptides and proteins assigned to contaminant proteins or reversed sequences were removed from the results, as were peptides not uniquely assigned to one protein. Peptides were grouped by protein IDs, and peptide abundances were normalized to the total ion count within each TMT channel. For expression (FITExP) and solubility (PISA) data sets in the method development part of the study, fold changes were calculated directly from protein abundances. For the validation data sets obtained by partial digestion (AFDIP^11^ and HOLSER^12^), fold changes were calculated from the abundances of top four peptides.

In protein-level analysis, molecules identified with modified peptides only were filtered away. In both analysis types, identifications with more than one missing value among the biological replicates (n ≥ 3) per condition (treatment or control) were removed, as well as proteins with fewer than two peptides. In the PISA-Expression data set, any remaining missing values were imputed using the average normalized abundance in other replicates. Protein lists from both analysis types were cross-validated, and only proteins present in both lists were retained to ensure fair comparison.

### Statistical Testing

For each peptide and protein, two-tailed, unpaired Student’s *t* test was performed comparing normalized abundances of treated versus control samples. Fold changes were calculated as the ratio of the median (among the replicates) abundances in treated samples to control samples. For PISA, expression, and OPTI-PISA data, only peptides with consistent fold-change direction were retained. Benjamini–Hochberg (BH) multiple testing correction was applied, and the resulting adjusted p-values were used in downstream analysis.

In peptide-level analysis, peptides belonging to the same protein ID were ranked by their p-value, fold changes or a composite score defined as −log10­(p-value) × |log2­(fold change)|. In Fisher’s method, either all peptides or the top N peptides (N = 2, 3, ... 6) for each protein were used. When N exceeded the number of available peptides for a given protein, missing values were handled in one of the two ways: (i) using only the available p-values (“no imputation”), or (ii) imputing the average p-value of the existing peptides for the same protein.

### Fisher’s Method of P-Value Aggregation

For each protein, X^2^ value was calculated from the p-values of N selected peptides:

1 $$X^{2}=-2\overset{\left\{N\right\}}{\underset{\left\{i=1\right\}}{\sum }}\ln p-{\mathrm{value}}_{i}$$

The combined protein p-value was computed as the one-tailed probability of observing a X^2^ value equal to or greater than the observed X^2^. This was done using the pchisq­() function in R.

### Figure-of-Merit for Evaluation of Strategies

The Figure of Merit (FoM) designed to determine which variation of Fisher’s approach, if any, is optimal, was based on the combined ranking of known targets of nine drugs (Supplementary Table 1). The selected drugs bind to a broad spectrum (106 in total) of experimentally validated human proteins registered as targets in DrugBank, and trigger diverse well-studied pathways, providing an objective benchmark for this study. The basic requirement for FoM was that analytical strategies that rank known targets higher (lower Rank value) should produce larger FoM values. For each drug but staurosporine, FoM was calculated as

2 $$\mathrm{FoM}=\overset{n}{\underset{k=1}{\sum }}\sqrt{\frac{1,000,000}{\mathrm{Rank}F_{k}}}$$

Here, RankFk—ranking factors of identified known targets (from DrugBank^13^), and n is the number of such targets. Ranking factors were calculated as follows: for each protein, a score was computed by combining the fold change of protein abundance with its associated p-value, obtained either from BH-corrected *t* tests (protein-level analysis) or from Fisher’s method (peptide-level analysis). The score was defined as −log10­(p-value) × |log2­(fold change)|. In the HOLSER^12^ and AFDIP^11^ data sets, fold changes of proteins were calculated either as the average fold changes or center of gravity shifts of the top N peptides, where the direction (positive or negative) was determined by the majority of selected peptides. Proteins were sorted by their scores in the descending order, and the obtained ranks were then rescaled to a uniform range of 1–8000 to enable comparison of data sets with different proteome depths. These rescaled ranks represented the ranking factors in (2). For staurosporine, a broad-spectrum kinase inhibitor with hundreds of kinases known to bind the compound, we counted the number of kinases within the top 100 ranked proteins, which served as the FoM score.

Lastly, the FoM values for individual data sets were renormalized for the same N to a 1–10 scale, and the rescaled values were summed together to obtain the final score for a given strategy.

### Data Sets Used for Method Evaluation

The proteomics data sets are listed in Table 1, and all known targets of drugs used in this study are given in Supplementary Table 1.

**Table 1:** Datasets Used for the Fisher’s Method in Proteomics Data Analysis

Name	Cell line	Proteomics assay	TMT sets	Search engine	Number of peptides after cleaning	Number of proteins after cleaning
**Data sets for method establishment**						
ProtargetMiner_MCF7 deep^8^	MCF-7	FITExP	3	PD	93102	9210
				MaxQuant	81860	8919
ThermoTargetMiner^14^	A549	PISA in lysate	3	PD	87139	6480
				MaxQuant	61837	6237
OPTI-PISA^10^	A549	OPTI-PISA in lysate	1	PD	81097	6562
				MaxQuant	65818	6184
OxidoResist_5-FU	SW480	PISA-Expression	1	PD	83949	7346
				MaxQuant	66310	6863
**Data sets for method validation**						
AFDIP^11^	HeLa	AFDIP	1	PD	66924	6940
HOLSER^12^	HeLa	HOLSER	1	PD	181529	8750

The first data set used for testing Fisher’s-based approach consisted of expression (FITExP) proteomic data from drug-treated MCF-7 cells extracted from the ProTargetMiner database.^8^ Briefly, MCF-7 cells were exposed for 48 h to IC_50_ concentrations of nine anticancer compounds representing diverse mechanisms of action. For FoM calculations, we used the known targets of bortezomib (PSMB1 and PSMB5) and raltitrexed (TYMS and FPGS). The second data set was a solubility (PISA^9^) assay from the ThermoTargetMiner database^14^ and originated from A549 cell lysate treated with different drugs against lung cancer. For FoM determination, we used the known targets of vorinostat (targets: HDACs), everolimus (target: MTOR) and olaparib (targets: PARP1, PARP2, and AKR1C3). The third data set was an automated PISA assay (OPTI-PISA^10^) obtained from A549 cell lysates treated with 10 μM methotrexate (MTX, target: DHFR), staurosporine (targets: kinases), or ganetespib (targets: HSP90s). The last data set comprised the combined PISA-expression^15^ proteomics profile of 5-fluorouracil (5-FU)-resistant versus 5FU-sensitive SW480 cells, with the 5FU targets of TYMS used for FoM calculations.

The data sets used for validation of the optimal Fisher’s-based strategy encompass data on protein domain accessibility to trypsin in the AFDIP^11^ and HOLSER^12^ techniques. In both cases, known targets of MTX (target: DHFR), staurosporine (targets: kinases), and rapamycin (targets: MTOR and FKBPs) were used for FoM determination.

## Results

As the majority of proteins in most data sets were identified with six or more peptides (an example is shown in Figure 1A), the range of tested N values was limited to six peptides. In the discovery part of the study, we found by comparing the FoM-based scores (Figure 1B and Supplementary Table 2) that for all N, ranking modes and imputation strategies Fisher’s method provides significantly better performance compared to conventional protein-level analysis. That is, known targets universally received on average better ranking when protein p-values in chemical proteomics data were obtained by aggregating peptide p-values using Fisher’s formula.

**Figure 1:**
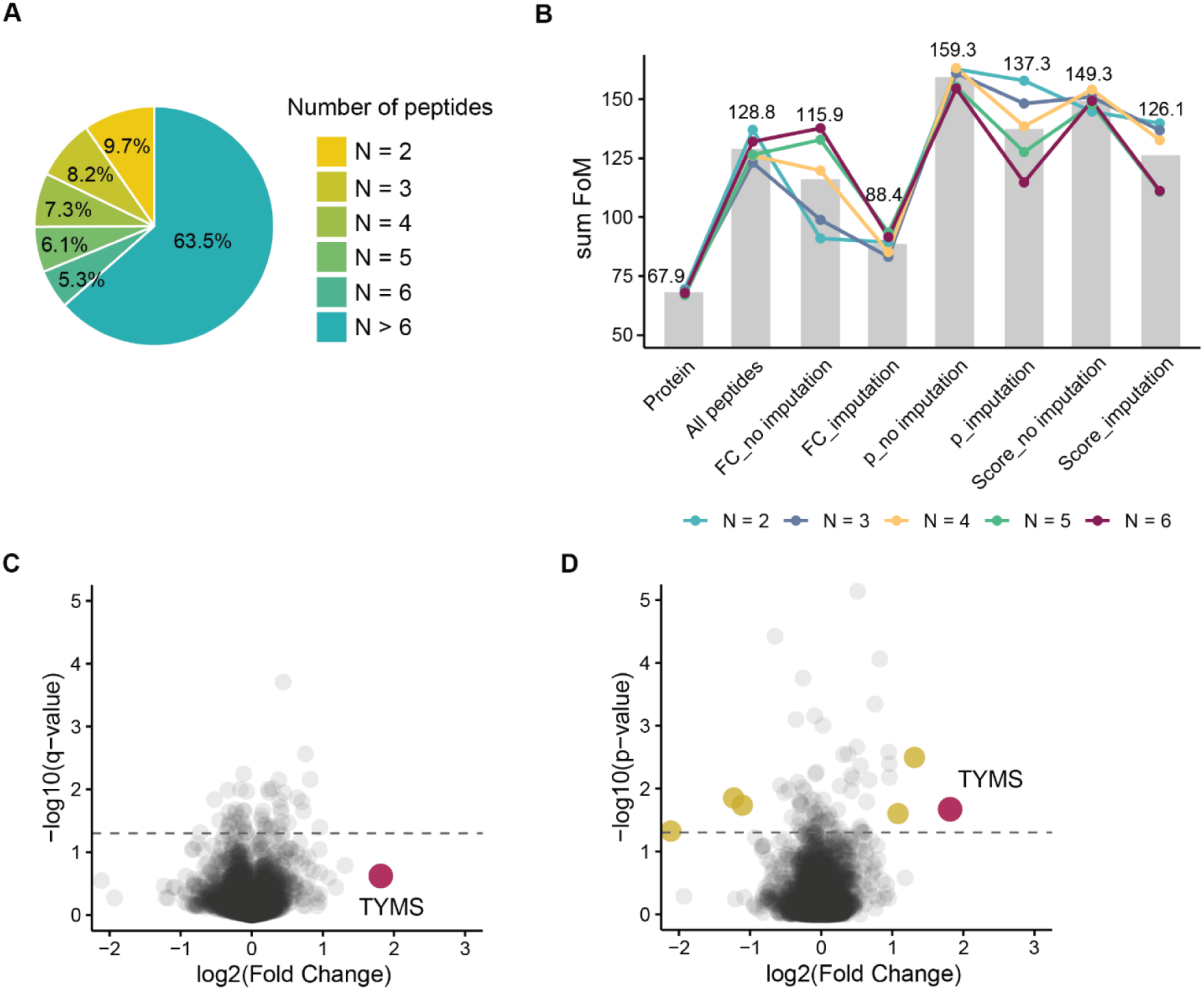
(A) Distribution of peptide coverage of proteins in the PISA-Expression data set of 5-FU resistant versus sensitive SW480 cells. (B) Comparison for different Fisher’s-based approaches of the summed figure of merit (FoM) values calculated as described in the text. (C) Raltitrexed’s target TYMS in the ProTargetMiner data set did not pass the significance threshold based on protein expression changes. (D) Fisher’s method applied to the top four peptides per protein ranked by Student’s *t* test p-value identified the known target of raltitrexed, TYMS, highlighted in purple. Other possible candidates are shown in yellow.

A closer look showed that incorporating imputed p-values when the number of available peptides was lower than N consistently reduced the analysis quality. Ranking peptides by their p-value obtained from *t* test was found to be the best, followed by ranking by a combination of p-value and fold change. Note that this ranking refers to the selection of peptides for Fisher’s analysis, while after protein p-value calculation the final protein ranking was done by combined score as described in the Methods.

Including in the analysis all available peptides for each protein was somewhat problematic because proteins with a larger number of peptides tended to yield artificially small aggregated p-values, introducing bias toward highly covered proteins. Therefore, detailed investigation was performed on the effect of N on the FoM values. Using top four peptides ranked by Student’s *t* test p-value yielded the most accurate target identification.

As an example, in the ProTargetMiner deep-proteome data set, the raltitrexed target TYMS ranked ninth in the protein-level analysis and did not even reach the p-value threshold of 0.05. In contrast, applying Fisher’s method to the top four peptides ranked by the p-value elevated TYMS to the top position by the combined score (Figure 1C and D).

To validate the developed Fisher’s-based approach, we applied it to detecting ligand-induced conformational changes by partial digestion (AFDIP^11^ and HOLSER^12^). Unlike the expression and solubility proteome profiling (FITExP^7^ and PISA^9^), where peptides belonging to the same protein behave the same way, in partial digestion only peptides related to drug binding change their abundance. Therefore, we expected that Fisher’s approach with limited N will be particularly suitable in these techniques compared to the traditional protein-based fold-change approach. A case in point is presented in Figure 2A and B, where none of the known rapamycin targets (MTOR and FKBPs, shown in purple) ranked among the top five proteins in the target candidate list obtained by conventional analysis. The top protein, CBR1, has no established connection to MTOR signaling. In contrast, using Fisher’s approach to the top four peptides with the lowest p-values as well as their fold changes as protein fold change revealed significant allosteric shifts for FKBP2 and FKBP3 (Figure 2C and D).

**Figure 2:**
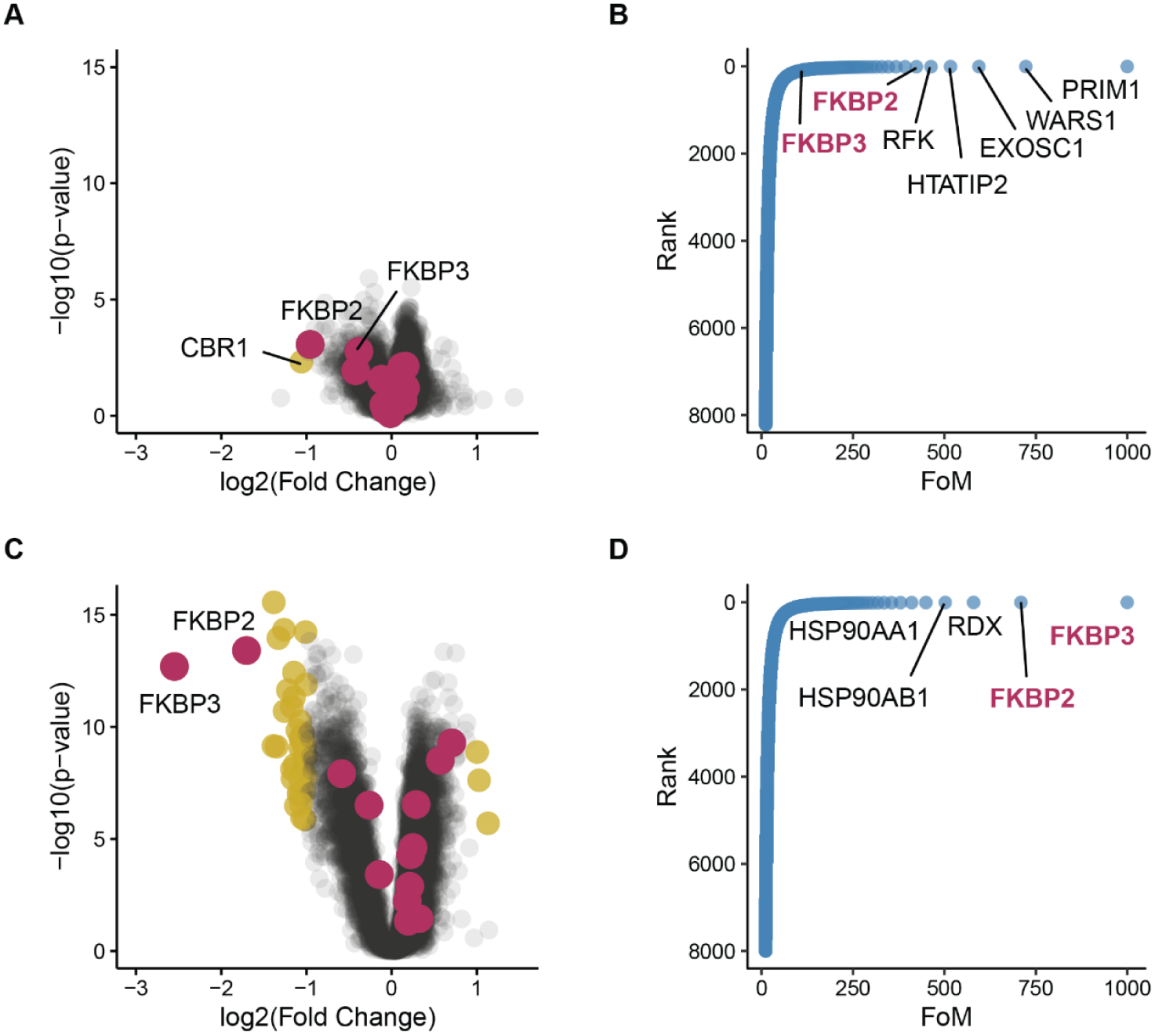
Comparison of rapamycin target identification in the HOLSER data set using either protein abundance data (A and B) or Fisher’s method applied to the four peptides with the smallest p-values without imputation (C and D). In the volcano plots (A and C), significance cutoffs for fold changes are ±1, and for p-value is 0.05. Significant proteins are shown in yellow, while known rapamycin targets (FKBPs and MTOR) are highlighted in purple. In waterfall plots (B and D), the vertical axis represents the rescaled rank that is based on a combination of fold change and p-value, and the horizontal axis represents each protein’s contribution to the FoM. Note that based on the protein abundance data (B), the known targets FKBP2 and FKBP3 are on the sixth and 110th positions, respectively, while they are the first and second targets using the optimal Fisher’s method applied on peptide abundance data (D).

A similar advantage was observed in the AFDIP peptide-level data set.^11^ Here, center of gravity differences ΔCoG between 1 treated and control 8 h digestion curves were quantified. While conventional approach did not produce any significant candidates after B.-H. correction, applying Fisher’s method to calculate protein p-value from top four peptides and averaging their ΔCoG values to represent ΔCoG of a protein correctly identified four known rapamycin targets—FKBP2, FKBP3, FKBP4, and MTOR—on the top of the ranked protein list (Figure 3).

**Figure 3:**
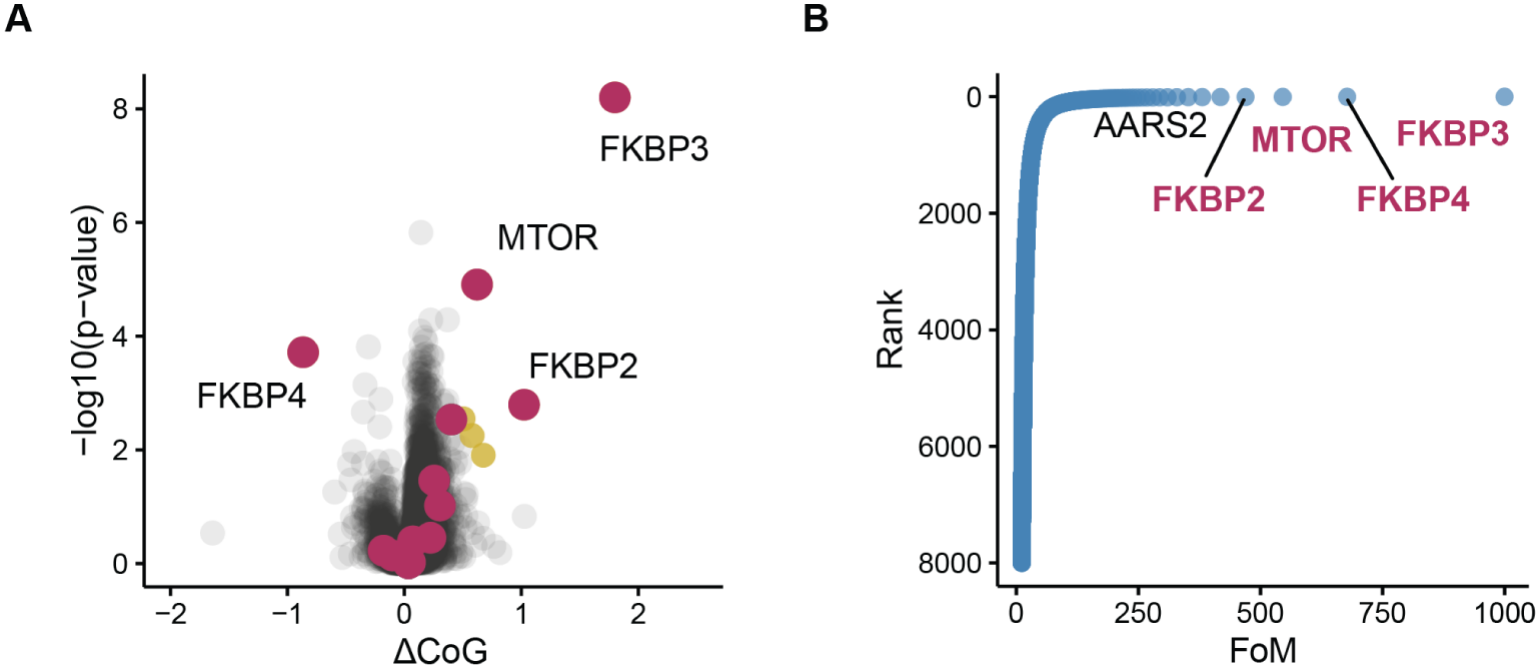
Application of Fisher’s method for rapamycin target identification in AFDIP data. Fisher’s method was applied using the top four peptides per protein, ranked by p-value and analyzed without missing value imputation. (A) Volcano plot. (B) Waterfall plot as in Figure 2B,D for the optimal Fisher’s method applied to peptide-level abundances. Note that known rapamycin targets (FKBP2, FKBP3, FKBP4, and MTOR) occupy the top four positions.

## Discussion

The results presented above demonstrate that aggregating peptide-level statistical evidence provides a more sensitive and robust strategy for protein-level inference in chemical proteomics than conventional protein abundance-based analysis. By operating directly on peptide-level p-values, Fisher’s method preserves heterogeneity among peptides belonging to the same protein and avoids dilution of true signals caused by noisy or uninformative peptide measurements. This effect was consistently observed across data sets representing protein expression, solubility, and ligand-induced conformational changes, indicating that the improvement is not assay-specific but reflects a general analytical advantage.

Although Fisher’s method formally assumes independence among combined tests, peptide-level measurements originating from the same protein are not strictly independent in all cases. Nevertheless, the consistent performance gains observed across diverse data sets, search engines, and proteomics platforms suggest that the approach is empirically robust to moderate peptide dependence. Importantly, restricting the aggregation to a limited number of top-ranked peptides further mitigates potential bias arising from peptide redundancy and shared technical variation.

A key observation of this study is that including all peptides associated with a protein can introduce systematic bias, as proteins with higher peptide coverage tend to yield artificially small aggregated p-values. Limiting the analysis to the top N peptides ranked by statistical significance effectively controls this bias while retaining sensitivity to true effects. Across all evaluated data sets, using the top four peptides provided the most reliable balance between robustness and sensitivity, highlighting peptide selection as a central component of accurate protein-level statistical inference.

Handling missing peptide-level information was found to be equally critical. Imputation of missing p-values consistently reduced analytical performance, likely due to artificial inflation of statistical evidence. In contrast, restricting Fisher’s aggregation to observed peptide-level p-values yielded more accurate and reproducible protein ranking, emphasizing the importance of conservative missing-value handling in peptide-based statistical frameworks.

The advantages of peptide-level p-value aggregation were particularly pronounced in partial-digestion-based assays, where only a subset of peptides proximal to ligand-binding sites is expected to respond to treatment. Under such conditions, conventional protein-level aggregation is inherently suboptimal, whereas Fisher’s method effectively captures localized peptide responses. As a downstream data analysis strategy, the proposed approach requires no changes to experimental design or quantification workflows and can be readily integrated into existing proteomics pipelines.

## Conclusion

In this study, we demonstrated that Fisher’s method provides a simple yet powerful statistical framework for integrating peptide-level information into protein data in proteomics analysis workflow. Instead of aggregating into a single protein abundance the corresponding peptide abundances and then performing statistical testing at the protein level, we conducted statistical testing at the peptide level and subsequently combined the p-values of the most informative peptides using Fisher’s method. By prioritizing peptides that exhibit significant treatment-induced changes and reducing the influence of noisy and/or uninformative measurements, this approach generates protein-level statistical evidence that more faithfully reflects true biological regulation.

Across multiple data sets representing protein expression, solubility, and conformational changes, Fisher’s method consistently outperformed conventional protein-level analyses. These findings establish Fisher’s method as a robust and generalizable strategy for improving identification of significantly changing proteins across diverse proteomics platforms.

## Supplementary Material



## Data Availability

The raw mass spectrometry proteomics data and search/quantification results are available with the Proteome change Consortium via the PRIDE repository under accessions: ProtargetMiner PXD013134, ThermoTargetMiner PXD054158, OPTI-PISA PXD050241, PISA-Expression PXD071434, HOLSER PXD069119, and AFDIP PXD061498.

## Notes

Table S1: summary of drugs and their validated targets utilized for Figure of Merit (FoM) calculations in both method development and validation; Table S2: scores of different analysis strategies (PDF)

## Abbreviations

TMT: Tandem mass tag
MQ: MaxQuant
PD: Proteome Discoverer
BH: Benjamini–Hochberg
FoM: Figure of Merit
