# Supplementary material for: Peptide-to-Protein Data Aggregation Using Fisher’s Method Improves Target Identification in Chemical Proteomics
Source: Anal Chem. 2026 Apr 22;98(17):12596–602. doi: 10.1021/acs.analchem.5c08021 (PMC13150797; doi:10.1021/acs.analchem.5c08021)
Supplement: Supplementary file 1 [file ac5c08021_si_001.pdf]

Peptide-to-protein data aggregation using Fisher's method improves target identification in chemical proteomics

Supporting Information

Hezheng Lyu[1,2], Hassan Gharibi[1,3,4,5], Zhaowei Meng[1,3,4,5], Bohdana Sokolova[1],

Susanna Lundström[1,3,4], Xuepei Zhang[1,3,4], Roman A. Zubarev[1,3,4,5,6]*

[1]Division of Physiological Chemistry I, Department of Medical Biochemistry and Biophysics, Karolinska Institutet, 171 65 Stockholm, Sweden

[2]Biomotif AB, 183 48 Täby, Sweden

[3]Swedish National Infrastructure for Biological Mass Spectrometry (BioMS), 17177 Stockholm, Sweden

[4]Chemical Proteomics Unit, Science for Life Laboratory (SciLifeLab), 171 65 Stockholm, Sweden

[5]Single Cell Proteomics facility, Department of Medical Biochemistry and Biophysics, Karolinska Institutet, 171 65 Stockholm, Sweden

[6]Department of Pharmaceutical and Toxicological Chemistry, Medical Institute, RUDN University, 6 Miklukho-Maklaya St, Moscow, 117198, Russia*Correspondence and requests for materials should be addressed to R.A.Z. (email: Roman.Zubarev@ki.se)

Table of contents

Supplementary Tables S1-S2 (page S2-S3)

Table S2. Drugs and known targets used for FoM calculation

Dataset Name	Drug name	Targets used for FoM calculation
ProtargetMiner_MCF7 deep	Bortezomib<br>Raltitrexed	PSMB5, PSMB<br>TYMS, FPGS
OPTI-PISA	MTX<br>Staurosporine<br><br><br><br><br><br><br><br><br><br><br><br><br><br><br><br><br><br>Ganetespib	DHFR<br>Kinases:<br>AAK1, TBK1, TNK2, MAP4K5, MYLK, MAP2K1, CAMK2G, STK24,<br>CDK2, CAMK2D, PHKG2, CAMKK1, CIT, CHEK1, AURKA, PDPK1, DAPK3, PIP5K1C, ROCK2, STK10, PAK4, CHEK2, PRKAR1A, SRC, PRKCA, PRKACA, CSNK2A2, PRKACB, RPS6KB1, GRK2, MARK3, MAP2K2, CSK, PRKCI, MAPK9, MAP2K4, MAP2K3, GSK3A, GSK3B, CDK9, RPS6KA3, PRKX, MAP2K6, MAPK12, PRKAA2, CDK5, CDK16, CDK17, PRKCE,<br>PTK2, PRKCZ, PRKCD, STK4, PRKAA1, STK3, ROCK1, PRPF4B,<br>DYRK1A, CAMK1, STK38, RPS6KA1, TAOK1, TLK2, STK32C,<br>CAMK1D, BRSK2, STK35, PASK, CAMKK2, MKNK1, CDK19, STK33, SLK, TAOK3, BMP2K, IRAK4, CDK12, NLK, DAPK2, PKN2, PRKAG1<br>HSP90AA1, HSP90AA2P, HSP90AB4P, HSP90AA4P, HSP90AB1,<br>HSP90B1
ThermoTargetMiner	Everolimus<br>Vorinostat<br>Olaparib	MTOR<br>HDAC1, HDAC2, HDAC3, HDAC6, HDAC8<br>PARP1, PARP2, AKR1C3
OxidoResist_5-FU_PISA-Expression	5-FU	TYMS
HOLSER	MTX<br>Rapamycin<br>Staurosporine	DHFR<br>MTOR, FKBPs (FKBP1A,2,3,4,5,7,8,9,10,11, 14, 15, FKBPL)<br>Kinases:<br>STK25, GAK, PDPK1, DAPK3, SPAG9, JAK2, RPS6KA4, AK1, CDK1, PHKG2, FER, PRKCA, PRKACA, PRKACB, RPS6KB1, JAK1, CDK2, GRK2, MARK3, TYK2, AKT2, TTK, CSK, PRKCI,<br>GSK3A, RPS6KA3, MAP2K6, PLK1, DAPK1, PRKAA2, CDK5, PTK2, PRKCD, CDK18, STK4, PAK1, PAK2, STK3, CAMK2G, MELK, STK38, RPS6KA2, PKN1, PKN2, PKN3, MARK2, MAP4K3,<br>CAMKK1, PIP4K2C, SLK, IRAK4, MARK1, STK26, TBK1, BAZ1B,<br>PACSIN3, STK38L, MAP4K5, STK24, ROCK2, WEE1, AXL, PRKAA1, PRPF4B, CDK9, STK17A, PAK4, CSNK1D, TNK1
AFDIP	MTX<br>Rapamycin<br>Staurosporine	DHFR<br>MTOR, FKBPs (FKBP1A,2,3,4,5,7,8,9,10,11, 14, 15, FKBPL)<br>Kinases:<br>CAMK2D, CHEK1, PHKG2, YES1, DAPK3, MAP3K7, PRKAR1A,<br>CDK4, PRKACA, RPS6KB1, CDK9, RPS6KA3, SRPK2, PRKCD,<br>PRKAA1, STK3, STK38, MARK2, CAMK1D, PIP4K2C, PHKB,

		EPS8L2, BMP2K, RIPK2, ROCK2, PAPSS2, CHEK2, PRKAR2A,<br>ADRBK1, AAK1, PDPK1, MAPK1, MAP2K2, PRKDC, HIPK1, RFK,<br>CHKB, SPAG9, EPS8, MARK3, TLK2, HGS, SMG1

Table S2. Scores of different analysis strategies

**Selection of N**	**Protein data**	**Peptide data + Fisher's method**						
		**All peptides**	**Top N by FC**		**Top N by p-value**		**Top N by score**	
			**no imputation**	**with imputation**	**no imputation**	**with imputation**	**no imputation**	**with imputation**
N = 2	69.3	136.9	90.8	89.2	162.7	157.8	144.7	139.7
N = 3	68.5	123	98.7	83	161	148.1	150.9	136.7
N = 4	67.2	126	119.6	85	163.1	138.4	154	132.5
N = 5	66.7	126.3	132.7	93.3	155.2	127.5	147.6	110.7
N = 6	67.6	131.9	137.6	91.4	154.5	114.6	149.2	111
sum	*339.3*	*644.1*	*579.4*	*441.9*	*796.5*	*686.4*	*746.4*	*630.6*
